# The liver transcriptome of two full-sibling Songliao black pigs with extreme differences in backfat thickness

**DOI:** 10.1186/2049-1891-5-32

**Published:** 2014-06-06

**Authors:** Kai Xing, Feng Zhu, Liwei Zhai, Huijie Liu, Zhijun Wang, Zhuocheng Hou, Chuduan Wang

**Affiliations:** 1 National Engineering Laboratory for Animal Breeding and MOA Key Laboratory of Animal Genetics and Breeding, Department of Animal Genetics and Breeding, China Agricultural University, 100193 Beijing, China; 2 Tianjin Ninghe Primary Pig Breeding Farm, Ninghe 301500, Tianjin, China

**Keywords:** Backfat thickness, Liver, Pig, RNA-Seq

## Abstract

**Background:**

Fatness traits in animals are important for their growth, meat quality, reproductive performance, and immunity. The liver is the principal organ of the regulation of lipid metabolism, and this study used massive parallelized high-throughput sequencing technologies to determine the porcine liver tissue transcriptome architecture of two full-sibling Songliao black pigs harboring extremely different phenotypes of backfat thickness.

**Results:**

The total number of reads produced for each sample was in the region of 53 million, and 8,226 novel transcripts were detected. Approximately 92 genes were differentially regulated in the liver tissue, while 31 spliced transcripts and 33 primary transcripts showed significantly differential expression between pigs with higher and lower backfat thickness. Genes that were differentially expressed were involved in the metabolism of various substances, small molecule biochemistry, and molecular transport.

**Conclusions:**

Genes involved in the regulation of lipids could play an important role in lipid and fatty acid metabolism in the liver. These results could help us understand how liver metabolism affects the backfat thickness of pigs.

## Background

Consumer choice of pig meat is an important factor that dictates the principles of swine breeding worldwide. One aspect of this is the deposition of fat in the muscle and backfat, which is associated with growth rate, meat quality, and reproductive performance [1]. Backfat thickness is highly correlated with body fat radio, carcass cross-sectional fat area ratios, and intramuscular fat, so is a good indicator of fat deposition in pigs [2]. Therefore, the selection of backfat thickness using B-mode real-time ultrasound is a practical and economical method in pig breeding for increasing feeding efficiency, carcass value, and consumer acceptance of pork [3]. Because of the increasing problem of human obesity worldwide, it is beneficial to regulate fat deposition in pig breeding through molecular markers. Additionally, their similarity to humans in body size and other physiological/anatomical features, including their innate tendency to over consume food, means that pigs are a good animal model for studying obesity [4].

The extent of fat deposition can be determined by triacylglycerol synthesis and storage, lipid mobilization, and fatty acid oxidation [5]. The liver is one of the most important organs to regulate appetite and body weight in pigs, as well as playing a key role in regulating several metabolic processes [6]. In pigs, *de novo* cholesterol synthesis and fatty acid oxidation mainly take place in the liver [7]. Lipid hydrolysis in adipose tissue results in free fatty acid, which is combined with plasma albumin then transported to the liver for use as an energy source through oxidation [8].

RNA-Seq technology for transcriptome profiling has previously been used to explore the transcriptome of pig liver tissue. Such transcriptomes were recently compared between a full-sibling (full-sib) pair of F2 females from a White Duroc × Erhualian resource population with extreme phenotypes in growth and fat deposition [9]. Other comparisons include a backcross of two female groups (H and L) with extreme intramuscular fatty acid composition (25% Iberian ×75% Landrace) [10], and Duroc × F2 (Leicoma × German Landrace) cross pigs with divergent skatole levels in backfat [11]. Although several studies have previously attempted to identify the genes and pathways involved in fatty traits in the liver, to our knowledge, they either lacked sufficient RNA-Seq liver samples or did not take into account the effect of different genetic background noise when analyzing liver regulation.

The Songliao black pig is a Chinese domestic breed with powerful stress resistance and good reproductive and fat deposition capabilities. It is therefore a good model for studying fatty deposition. In the present study, we used RNA-Seq to obtain the liver transcriptomes of two full-sib Songliao black pigs with a high variation in backfat thickness. The main aim of this study was to elucidate the genes and pathways involved in lipid metabolism in liver tissue using RNA deep sequencing technology.

## Methods

### Experimental design, animals, and phenotypes

The Songliao black female pig population (average age, 217 (range, 216–218) days; average live weight, 100 kg (range, 92.5-116.4 kg) was housed in consistent and standard environmental conditions with natural, uncontrolled room temperature and light. Animals were fed three times a day and had access to water *ad libitum*. Pedigree information was available for all animals. Live backfat thickness was measured on the last 3/4 rib using B-mode real-time ultrasound (HS1500, Honda, Japan). We analyzed a total of 53 individuals with full/half-sibs for backfat thickness to identify pairs with two divergent phenotypes. To minimize the noise of different genetic back grounds, full-sibs were selected as a priority.

We set out to compare transcriptome changes between two groups with a high variation in backfat thickness: pigs with higher backfat thickness (BH) and those with lower backfat thickness (BL) which had a backfat thickness 2–3 times lower than that of BH pigs. The chosen animals also had to have a similar backfat thickness within the same group (BH/BL) after adjustment for live body weight. Based on our criteria, experimental samples were made up of two pairs of pigs with extreme backfat thickness differences, both of which were full-sibs.

The chosen pigs were slaughtered according to guidelines for the ethical use and treatment of animals in experiments in China. Liver tissue was separated and stored in liquid nitrogen until analyzed. Total liver RNA was extracted using the total RNA extraction Kit (Bioteke, China) according to the manufacturer’s recommendations. The quality of total RNA was assessed by the 2100 Bioanalyzer (Agilent, USA).

### mRNA library construction and sequencing

mRNA was isolated from total RNA samples using oligo (DT) magnetic beads (Invitrogen, USA). Purified mRNA was first fragmented by the RNA fragmentation kit (Ambion, USA), then a one paired-end library was prepared for each sample according to the manufacturer’s instructions. mRNA libraries were individually sequenced for foursamples (two from each of the BH and BL group) using the Illumina High-seq 2000 sequencing system. The libraries were sequenced using a multiplexed paired-ends protocol with 180 bp of data collected per run. The average insert size for the paired-end libraries was 180 bp. A total of four paired-end mRNA libraries were constructed individually for four liver samples.

### Mapping and counting reads

Quality control and reads statistics were determined using FASTQC (http://www.bioinformatics.babraham.ac.uk/projects/fastqc/). All reads were trimmed 20-bp from the 5′ end according to the reads quality distributions. After removal of the sequencing adapt and low-complexity reads, all RNA-Seq reads were mapped on the reference pig genome (Sscrofa10.2) using TopHat v2.0.1 software [12] with default parameters. The annotation database Ensembl Genes v67 was used as a reference. Additionally, the intersect from BED Tools was used to count the number of reads mapping to exons, introns, and intergenic positions in the genome [13]. The reads count was measured using easy RNASeq software [14] to quantify the raw reads mapped on each gene.

### Differential expression and novel transcript analysis

The trimmed mean of M-values (TMM) was used to normalize gene expression levels [15]. After normalization, the NOISeq package implanted in the R computation environment was used to detect differentially expressed genes (DEGs) between two groups [16]. This method infers the noise distribution from the data and performs pair wise comparisons of the samples to identify DEGs. To measure expression level changes between two conditions, NOISeq takes into consideration two statistics: M(the log_2_-ratio of the two conditions) and D(the absolute value of the difference between conditions). The probability thresholds were *P* ≥ 0.8 and the TMM value in the lower expressed sample was ≥1. The higher the probability, the greater the change in expression between the two groups. Using a probability threshold of 0.8 means that the gene is 4 times more likely to be differentially expressed than non-differentially expressed [17]. Novel transcripts, differentially expressed spliced transcripts, and primary transcripts were also detected using the Cufflinks suite of software for RNA-Seq [12].

### Functional enrichment analysis of differentially expressed genes

Because the pig genome is poorly annotated, pig gene IDs were converted to human gene IDs using BioMart. DEG lists were submitted to the Database for Annotation, Visualization and Integrated Discovery (DAVID) bioinformatics resource for enrichment analysis of the significant overrepresentation of GO biological processes (GO-BP), molecular function (GO-MF), cellular component (GO-CC), and KEGG-pathway category [18]. In all tests, *P*-values were calculated using Benjamini-corrected modified Fisher’s exact test and ≤0.05 was taken as a threshold of significance.

To further identify the DEG interaction network in the liver, the Search Tool for the Retrieval of Interacting Genes (STRING) was used, which is based on a known protein-protein interaction database program. It generates a network of interactions from a variety of sources, including different interaction databases, text mining, genetic interactions, and shared pathway interactions [19].

### Quantitative PCR and data analysis

Total RNA was extracted from the liver and converted into cDNA using the Revert Aid™ First Strand cDNA Synthesis Kit (Thermo Fisher Scientific Inc, USA) following the manufacturer’s protocol. cDNA samples were analyzed with real-time reverse transcriptase (RT)-PCR using the Light Cycler® 480 Real-Time PCR System (Roche, USA). RT-PCR reactions were performed in a final volume of 20 μl with the Roche SYBR Green PCR Kit (Roche) according to the manufacturer’s instructions. Pig *GAPDH* was used as an internal standard to correct the cDNA input. Triplicate RT-qPCRs were performed for each cDNA and the average Ct was used for further analysis. Relative quantification values were calculated using the 2^-ΔΔCt^ method.

### Data availability

Complete data sets have been submitted to NCBI Sequence Read Archive (SRA) under Accession no. SRP035376, Bioproject: PRJNA234465.

## Results

### Analysis of RNA deep sequencing data

The backfat thickness of the pig carcass and weight of the kidney were shown to differ greatly between groups (Table 1). In general, individuals in the BH group had twice the backfat thickness compared with those in the BL group. Our experimental population is a conserved breed that has not undergone extensive selection nor hybridized with other breeds. Individuals therefore have very similar genetic backgrounds.

**Table 1:** The traits of backfat thickness and related fat deposition

**ID**	**H710**	**H712**	**H906**	**H909**
Backfat thickness of live, mm	24.9	9.4	21.7	8.8
Backfat thickness of carcass, mm	37.0	18.9	31.7	15.5
Kidney fat, kg	1.45	0.7	1.5	0.75

H710, H712 and H906, H909 are two pair full-sibs pigs. H710, H906 and H712, H909 are the BH and BL groups respectively.

We obtained approximately 53 million paired-end clean reads of 90 bp for each sample, and high percentages of mapped reads ranging from 84.80 to 92.90%. The number of reads and percentages of mapped reads were similar between the two groups (Table 2). Most mapped reads were located within an exon, with percent ages ranging from 72.85 to 76.21%. Other reads mapped within the untranslated region, introns, and intergenic regions. The percentages of reads in each region are shown in Table 2.

**Table 2:** The number of reads obtained and percentages of mapped reads per sample

**Sample ID**^**1**^	**Total reads number, M**^**2**^	**Mapping rate, %**^**3**^	**CDS exons, %**	**5′UTR, %**	**3′UTR, %**	**Intron, %**
H906	53.08	84.80	72.85	1.78	10.43	6.45
H909	52.16	92.90	76.21	2.64	7.98	5.11
H710	54.88	87.86	75.85	1.81	9.77	5.01
H712	51.56	88.17	75.28	1.79	8.89	6.35

1. H710, H712 and H906, H909 are two pair full-sibs pigs. H710, H906 and H712, H909 are the BH and BL groups respectively.

2. Indicate millions of reads.

3. Use the Ensembl V68 as the reference genome annotation to classify the mapping tags into the different regions. Ratio of the tags mapping on the subregion of the gene was calculated as the tags on each region divided by the total tags on the whole genome.

The total number of genes expressed in the liver in the four samples ranged from 16,815 to 17,025 (Additional file 1: Table S1), with numbers of expressed genes being similar between the two groups. Correlations between biological replicate samples showed that the expressed genes were very highly reproducible, suggesting that a major fraction of the liver transcriptome is conserved between groups.

**Figure 1:**
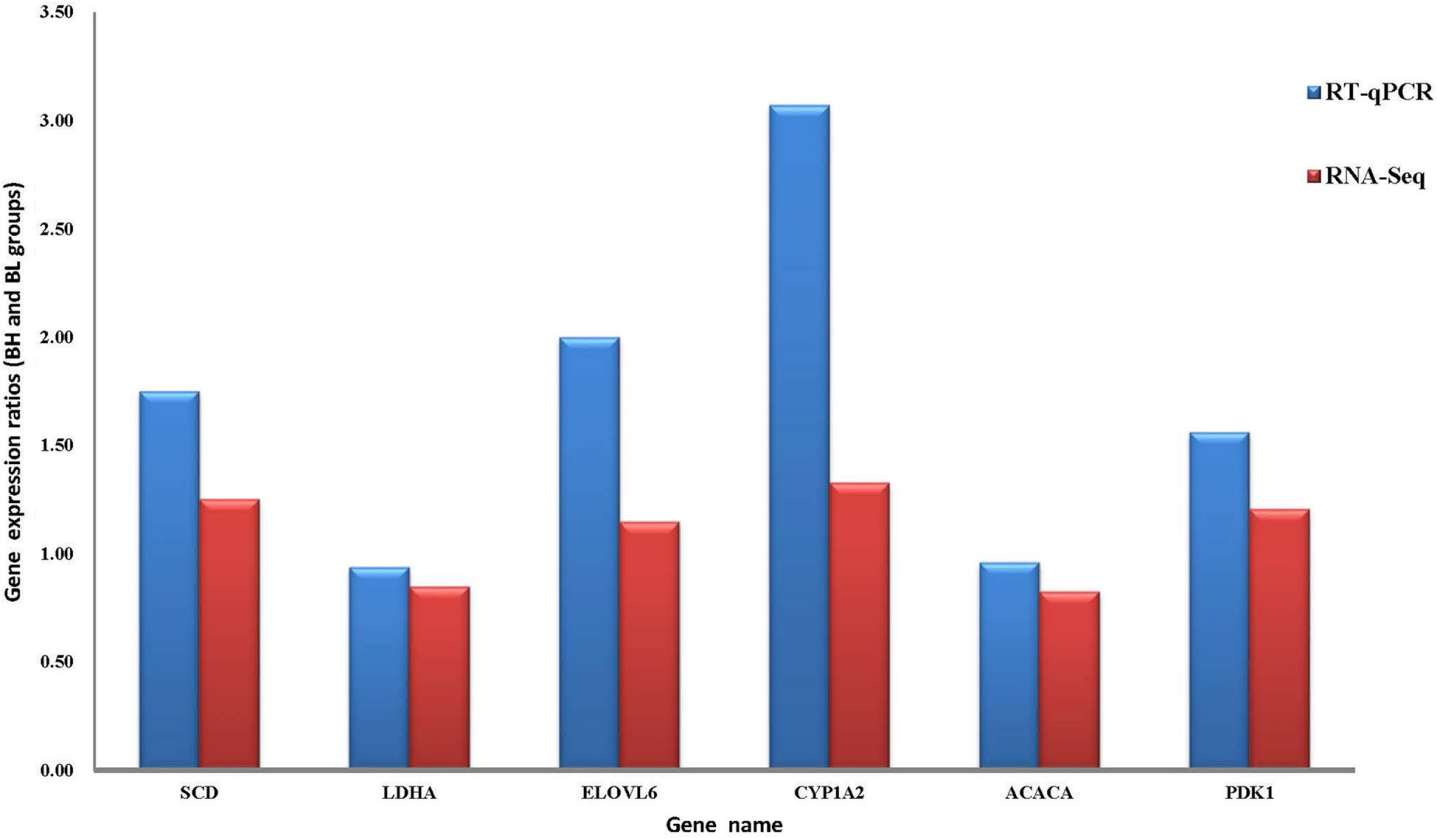
Comparison of qPCR and RNA-Seq expression ratios (BH and BL groups) for selected genes.

To confirm changes in transcript levels between BH and BL groups, six genes related to fatty acid synthesis or lipid metabolism were selected for RT-PCR analysis: *ACACA*, *LDHA*, *ELOVL6*, *CYP1A2*, *PDK1*, and *SCD*. We designed RT-PCR primers (Additional file 2: Table S2) for these genes, using *GAPDH* as a reference. When gene expression levels were compared, a strong correlation between RT-qPCR and RNA-Seq platforms was observed (0.72), confirming the high reproducibility of the data. For all six genes, the fold-change ratios between H and L groups were consistent with the RNA-Seq data (Figure 1).

### Differentially expressed genes between H and L groups

**Figure 2:**
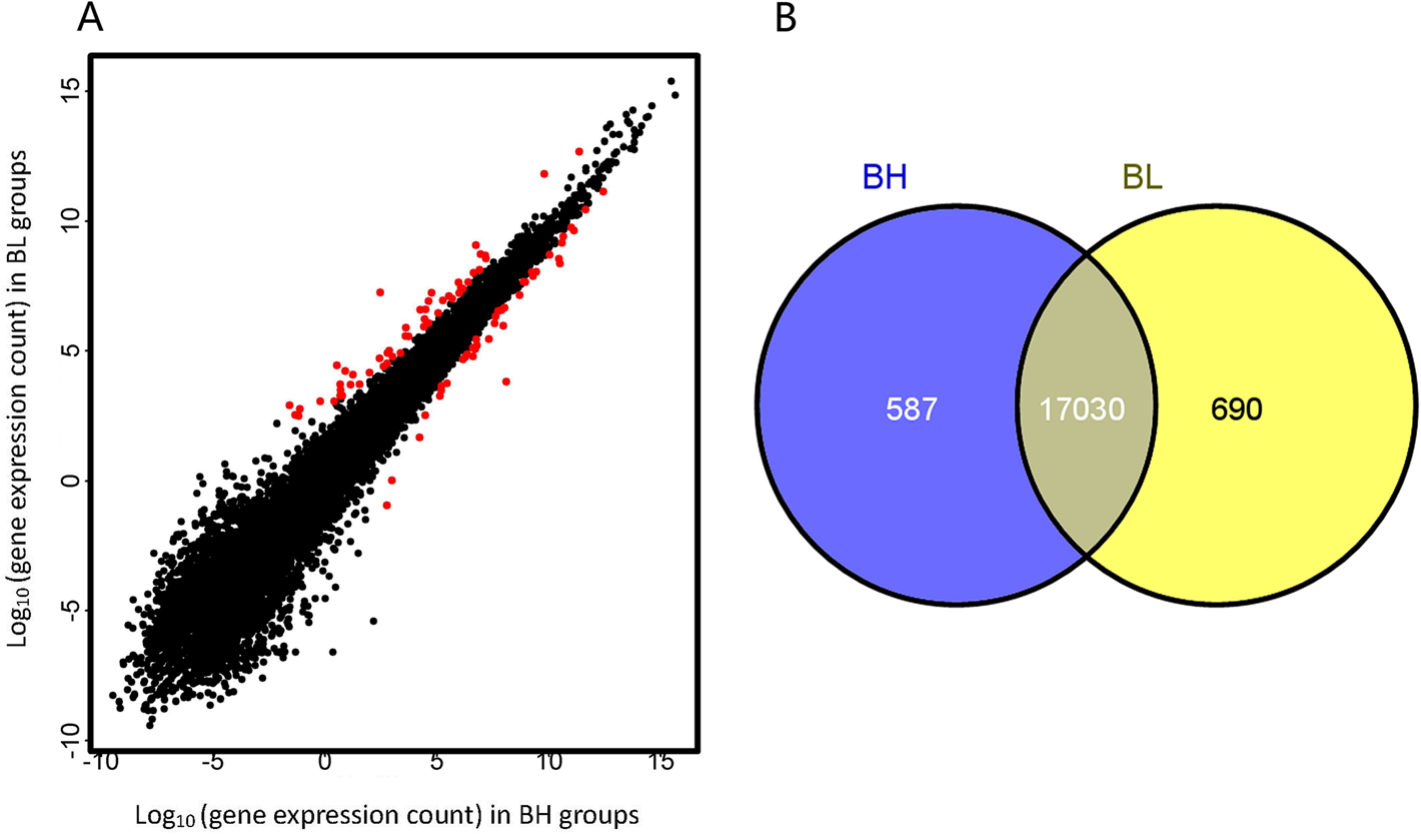
**Gene expression in BH and BL groups. A**. Red points represent genes that are significantly differentially expressed. **B**. Venn diagram showing genes only expressed in the BH group (blue circle), only expressed in the BL group (yellow circle), and common to both groups (intersection).

We quantified transcript expression levels in TMM to normalize gene expression data across different samples. Differential gene expression in liver tissue was calculated from the raw reads using the NOISeq package. We treated high/low backfat thickness samples as biological replicates because they showed similar phenotypes (Table 1). This process identified 92 liver DEGs between pigs with extreme high and low backfat thickness levels (Additional file 3: Table S3) We also identified 587 and 690 genes that were only expressed in the BH or BL group, respectively (Figure 2). Comparing shared DEGs between the two different pairs of pigs, we found that DEGs of biological replicates were more homogeneous with fewer false positives. Because of the sample limitations for each pair, we only present functional analysis of DEGs obtained by treating the two pig pairs as two biological replicates.

### Novel transcripts

We used the reference annotation based transcript algorithm implemented in Cufflinks to identify novel transcripts that were not annotated in the current Ensembl pig gene annotation database. A total of 8,226 novel transcripts were detected for FPKM ≥ 1 in the Songliao black pigs. Thirty-one spliced transcripts and 33 primary transcripts were significantly differentially expressed between the two groups (Additional file 4: Table S4).

### Functional enrichment analysis of the DEGs

Of the 92 DEGs, 39 were up-regulated and 53 were down-regulated in the BH group compared with the BL group. Pig gene IDs were converted to human gene IDs, but five genes did not match with their human homologs (ENSSSCG00000008012, ENSSSCG00000016695, ENSSSCG00000030368, ENSSSCG00000010427, and ENSSSCG00000012881).

**Figure 3:**
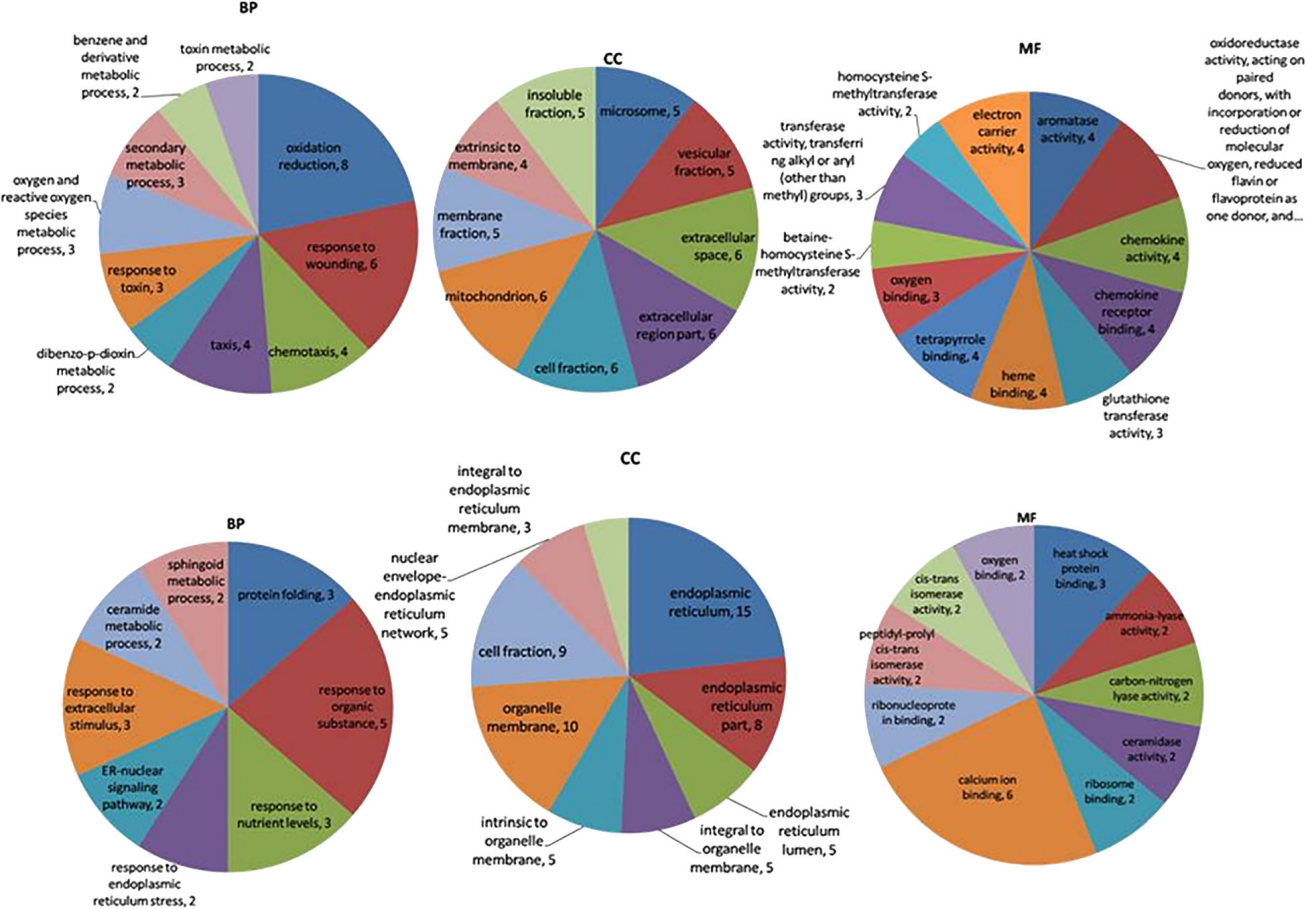
**DEG GO analysis in liver tissue.** Top three charts show gene ontology annotation processes (biological process (BP), cellular component (CC), and molecular functions (MF)) of up-regulated genes. Lower three charts show processes (BP, CC, and MF) of down-regulated genes.

To gain an insight into the liver tissue processes that differ between the BL and BH groups of pigs, differentially up-regulated and down-regulated genes underwent separate pathway analysis and gene ontology analysis using DAVID. Human homologs were recognized for 32 of the 39 up-regulated genes, and up-regulated genes were found to be involved in the metabolism of xenobiotics, drugs, retinol, and tryptophan (Table 3). The expression of DEGs in the enriched pathways is shown in Table 4. Following GO analysis, the DEGs were shown to be related to biological processes such as amino acid biosynthesis and metabolism, small molecule metabolism, and oxidation reduction. Forty-five of the 53 down-regulated genes had annotations in DAVID. No significant pathway was found to be associated with down-regulated genes. GO analysis showed that the biological processes enriched by DEGs are complex and relate to protein transport and enzyme activity (Figure 3).

**Table 3:** Pathways enriched in up-regulated genes in the liver (BH vs. BL)

**Pathway**	**Gene**	***P*** **value**	**Benjamini**
Metabolism of xenobiotics by cytochrome P450	CYP1A2, LOC100515394,LOC100526118, CYP1A1, LOC100511647, ADH1A CYP2S1	1.53E-08	4.43E-07
Drug metabolism	CYP1A2, LOC100515394, GSTM4 LOC100511647, ADH1A, CYP2A6	9.02E-07	1.31E-05
Retinol metabolism	CYP1A2, LOC100515394, CYP1A1 ADH1A, CYP2A6	1.88E-05	1.82E-04
Tryptophan metabolism	CYP1A2, CYP1A1, OGDHL	0.006753	0.047941

**Table 4:** Expression levels of genes in up-regulated pathways in the liver (BH vs. BL)

**Pathway**	**Gene symbol**	**BH**	**BL**	**Probability**
Metabolism of xenobiotics by cytochrome P450	CYP1A2	260.26	100.93	0.83
	LOC100515394	71.57	25.53	0.83
	LOC100526118	1539.43	574.36	0.85
	CYP1A1	81.60	29.08	0.83
	LOC100511647	3211.75	1402.03	0.81
	ADH1A	2055.82	869.66	0.82
	CYP2A6	1596.90	683.59	0.82
Drug metism	CYP1A2	260.26	100.93	0.83
	LOC100515394	71.57	25.53	0.83
	GSTM4	192.04	66.50	0.85
	LOC100511647	3211.75	1402.03	0.81
	ADH1A	2055.82	869.66	0.82
	CYP2A6	1596.90	683.59	0.82
Retinol metabolism	CYP1A2	260.26	100.93	0.83
	LOC100515394	71.57	25.53	0.83
	CYP1A1	81.60	29.08	0.83
	ADH1A	2055.82	869.66	0.82
	CYP2A6	1596.90	683.59	0.82
Tryptophan metabolism	CYP1A2	81.60	29.08	0.83
	CYP1A1	81.60	29.08	0.83
	OGDHL	6.83	0.52	0.82

Probability: The higher the probability, the greater the change in expression between conditions.

BH, BL: expression level in BH or BL after TMM normalization.

### Protein-protein interaction analysis

**Figure 4:**
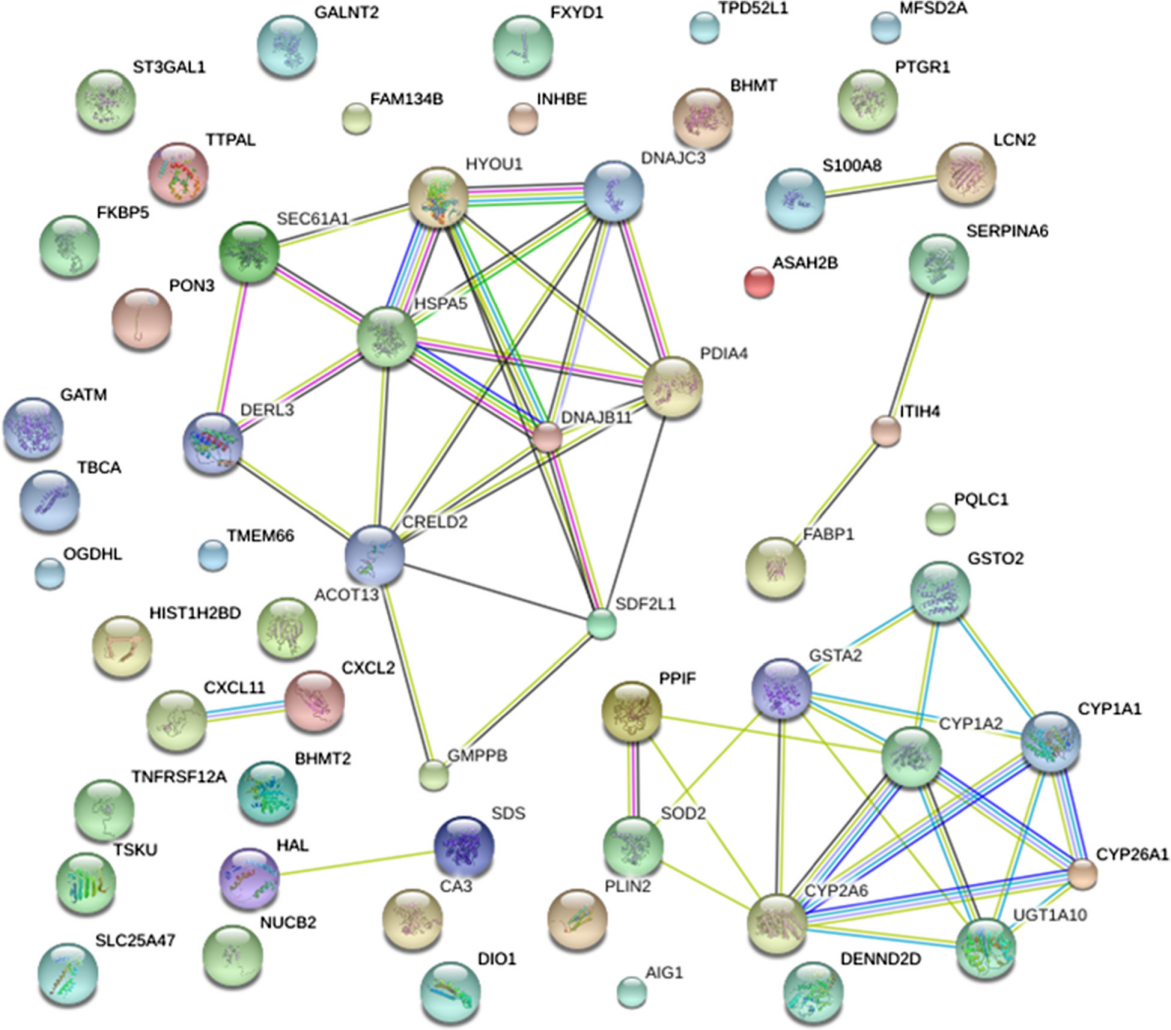
**STRING analysis shows that DEGs are involved in known and predicted protein-protein interactions.** STRING analysis of DEGs in the livers of BH and BL pigs. Network nodes represent genes shown in Additional file 2: Table S2. Lines of different color represent seven types of evidence used in predicting associations. Red line: fusion evidence; green line: neighborhood evidence; blue line: co-occurrence evidence; purple line: experimental evidence; yellow line: text mining evidence; light blue line: database evidence; black line: co-expression evidence.

To gain a better understanding of the biological relationships between genes, the integral DEG list was inputted into the STRING database. Most proteins encoded by DEGs were dissociative, and two protein-protein interaction networks were identified (Figure 4). In one network, genes were associated with metabolism, detoxification, and superoxides, while genes in the other network were related to the heat stress response.

## Discussion

In the present study, the percentages of mapped reads obtained per individual animal (84.80–92.90%) were higher than those in previous porcine liver transcriptome studies: 61.4–65.6% [9], 71.42–77.75% [10], and 43–84% [11]. Additionally, the percentage of reads within exons was higher than in previous studies, indicating that our results are more effective and credible.

Few studies have investigated how the liver transcriptome affects fat deposition. Our current gene expression analyses show that DEGs and pathways may play very important roles in this, and that genes involved in material metabolism and fatty acid transport are more active in the fatter groups of animals. These findings are consistent with those of previous studies. Previously, the expression levels of *CA3*, *SERPINA6*, *GATM*, *GSTA2*, and *ALAS1* were also found to be significantly different between the two groups [9]. *CA3* is associated with the internal fat rate and backfat thickness ofthe pig [20], while *SERPINA6* within the quantitative trait loci is associated with cortisol levels, fat, and muscle content, so can be considered a key regulator of obesity susceptibility [21]. These findings indicate that *CA3* and *SERPINA6* are strongly associated with fatty traits.

It is noteworthy that several DEGs identified in the present study (*FABP-1*, *LCN2*, *PLIN2*, *CYP1A1*, *CYP1A2*, *CYP2A6*, and *CYP26A1*) are involved in lipid metabolism. As a member of the family of fatty acid-binding proteins, *FABP-1* was expressed in all 12 tissues studied in a previous investigation, but transcript levels were more abundant in the liver and small intestine [22]. FABP-1 is involved in the transport of fatty acids to the site of β-oxidation, as well as the synthesis of triacylglycerol and phospholipids. It is also shown to interact with peroxisome proliferator-activated receptor (PPAR)α, which regulates fatty acid catabolism [23]. Compared with leaner pigs, *FABP* mRNA and protein levels were expressed at higher levels in the muscle of fatter animals [24]. In the present study, *FABP-1* was up-regulated in the liver samples of BH pigs compared with BL. Consistent with our results, *FABP-1* was also up-regulated in obese ob/ob mice compared with controls [25], suggesting that it’s increased expression may enhance fatty acid transport.

The adipokine lipocalin 2 (LCN2) is a member of the lipocalin family that transports small lipophilic ligands and is highly expressed by fat cells both *in vitro* and *in vivo*[26]. Levels of *LCN2* mRNA are dramatically increased in the adipose tissue and liver of ob/ob mice, while reducing *LCN2* expression leads to a decrease in *PPARγ* expression [27]. However, we found that *LCN2* was down-regulated in the BH group compared with the BL group of pigs. Perilipin 2 (PLIN2) is a cytosolic protein that promotes the formation and stabilization of intracellular lipid droplets, which are organelles involved in the storage of lipid depots. *PLIN2* polymorphisms have been associated with carcass traits including backfat thickness in pigs [28], while increased *PLIN2* mRNA expression was detected in the skeletal muscle of pigs with higher intermuscular fat [28,29]. Our results contradict this by revealing a lower *PLIN2* mRNA expression level in the livers of pigs with higher backfat thickness. Finally, *CYP1A1, CYP1A2*, *CYP2A6*, and *CYP26A1* belong to the highly diverse CYP450 super family, and also showed a differential expression pattern in the current study. CYPs have been shown to play critical roles in catalyzing metabolism reactions and in the oxidation of unsaturated fatty acids [30]. Because several differences appear to exist in observed expression levels between studies, further investigation into expression differences of these genes is warranted to elucidate their role in fat deposition.

The present study identified more pathways that were up-regulated than down-regulated in the fatter group of pigs. Most of these pathways are related to the metabolism of substances such as xenobiotics, drugs, retinol, and tryptophan. The retinoid metabolism pathway has previously been shown to be up-regulated in the liver. Retinoid is crucial for most forms of life, and many studies have identified an association between retinoid and lipid metabolism [31]. Retinoids regulate metabolism by activating specific nuclear receptors, including the retinoic acid receptor and the retinoid X receptor, an obligate heterodimeric partner for other nuclear receptors including PPARs. This helps to coordinate energy balance [32] and suggests that the liver has a larger burden in fatter compared with leaner pigs.

STRING analysis in the present study indicated that genes regulating metabolism influence those related to heat stress. Heat stress was previously shown to affect lipid peroxidation, causing serious damage to membrane lipids, lipoprotein, and other lipid-containing structures [33]. In pigs, body weight is positively correlated with heat stress [34], but our current results show that mRNA levels of genes related to heat stress are down-regulated in the livers of fatter compared with leaner pigs. Future investigations into the interaction between body weight and heat stress may therefore identify novel methods to show how body weight affects healthy individuals.

## Conclusions

This study undertook transcriptome analysis between two groups of Songliao black pigs with different backfat thicknesses. A total of 92 DEGs were identified between BH and BL groups. In concordance with the phenotypic differences, these genes belonged to pathways and gene networks related to lipid metabolism, regulation, and transport. Additionally, the identified DEGs related to heat stress could provide a new method of understanding and combating obesity. Our findings will be of use in understanding liver lipid regulation and in the design of new selection strategies to improve pig production.

## Abbreviations

BH: Group with higher backfat thickness; BL: Group with lower backfat thickness; DAVID: Database for annotation, visualization and integrated discovery; DEG: Differentially expressed gene; Full-sib: Full-sibling; GO-BP: Gene ontology biological processes; GO-CC: GO cellular component; GO-MF: GO molecular function; LCN2: Lipocalin 2; PLIN2: Perilipin 2; PPAR: Proliferator-activated receptor; TMM: Trimmed mean of M-values; RT: Reverse transcriptase; STRING: Search tool for the retrieval of interacting genes.

## Competing interests

The authors declare that they have no competing interests.

## Authors’ contributions

KX carried out the experiment and drafted the manuscript. ZCH and CDW conceived the study, participated in its design and coordination, and helped draft the manuscript. FZ, HJL and ZJW helped sample, experiment and analysis of data. All authors read and approved the final manuscript.

## Supplementary Material

Additional file 1: Table S1 Gene expression count in four samples. Click here for file

Additional file 2: Table S2 Primer sequences of six genes related to lipid metabolism for qRT-PCR. Click here for file

Additional file 3: Table S3 Liver DEGs between BH and BL pigs with biological replicates. Click here for file

Additional file 4: Table S4 Differentially expressed spliced transcripts and primary transcripts. Click here for file
